# Impact of QTL number, heritability and reference population size on the benefit of machine learning models over GBLUP in genomic prediction

**DOI:** 10.1186/s12711-026-01065-6

**Published:** 2026-07-13

**Authors:** Jifan Yang, Yvonne C. J. Wientjes, Mario P. L. Calus, Pascal Duenk

**Affiliations:** Animal Breeding and Genomics, Wageningen University & Research, Wageningen, 6700 AH The Netherlands

## Abstract

**Background:**

With the successful application of machine learning (ML) in many areas, its potential for genomic prediction (GP) in animal and plant breeding has attracted growing attention. However, despite numerous studies, the benefit of ML models over GBLUP remains controversial. Some studies reported higher accuracy of ML than linear models with small reference populations, but this benefit often disappeared in larger datasets. Additionally, the number of QTL also affects the prediction accuracy of ML models. The aim of this study was to compare the performance of three ML models, random forest (RF), support vector regression (SVR), and multilayer perceptron with residual networks (MLP-ResNet), with that of GBLUP. We simulated livestock populations with different data characteristics, including heritability, reference population size, and the number of QTL underlying the trait.

**Results:**

Our results showed that the number of QTL strongly affected the prediction accuracy of RF and MLP-ResNet, but had little effect on GBLUP and SVR. The accuracy of RF dropped markedly with increasing QTL number, whereas the accuracy of MLP-ResNet decreased with increasing QTL number when reference populations exceeded 10,000. Both RF and MLP-ResNet achieved benefits over GBLUP only when the number of QTL was small (i.e. ≤ 100). RF outperformed GBLUP with small reference populations, whereas MLP-ResNet outperformed GBLUP with large populations. SVR showed marginally lower accuracy than GBLUP across scenarios and required more computation time. Among all models, GBLUP showed the lowest dispersion bias.

**Conclusions:**

RF and MLP-ResNet achieved higher accuracy than GBLUP only when the number of QTL was small, whereas in all other situations GBLUP outperformed the ML models. The benefit of ML models over GBLUP occurred mainly in scenarios where the infinitesimal model assumption of GBLUP did not hold. Our results suggest that when introducing a new GP model, particularly tree-based or neural network methods, it is essential to evaluate its performance on simulated datasets across different numbers of QTL and reference population sizes. These findings provide insights for applying ML in animal and plant breeding.

**Supplementary Information:**

The online version contains supplementary material available at 10.1186/s12711-026-01065-6.

## Background

Genomic prediction (GP) has been widely implemented in animal breeding programs to predict genomic breeding values of animals [1–4]. At the moment, linear models, such as genomic best linear unbiased prediction (GBLUP) and Bayesian methods, are commonly used in GP [5, 6]. These models assume that the genotype–phenotype relationship is defined by a finite set of parameters that determine the distribution and variance structure of SNP effects. For example, GBLUP assumes that SNP effects are normally distributed with a common variance, which is one of the parameters in the model [6, 7]. In contrast, machine learning (ML) models have few or no specific assumptions about the distribution of the effects of the features (SNPs), offering greater flexibility in capturing complex relationships between genotypes and phenotypes [8–10]. Therefore, it is appealing to investigate the use of ML methods for GP.

Commonly used ML models in GP can be broadly classified into three categories based on their algorithmic structures, namely ensemble methods, kernel methods and deep learning. Random forest (RF) is an ensemble method that builds multiple decision trees using bootstrapped datasets and random subsets of features, and aggregates their predictions [11]. Support vector regression (SVR) is a kernel-based method that constructs a function in a high-dimensional feature space using support vectors and kernel functions [12]. Multilayer perceptron (MLP) is a type of feedforward artificial neural network consisting of an input layer, one or more hidden layers, and an output layer, trained by backpropagation [13]. Each ML method has its own strengths and limitations, with varying applicability depending on data structure and prediction objectives.

Some studies have reported that for GP, ML models outperformed linear models in terms of prediction accuracy, calculated by the correlation between predicted values and phenotypes [10, 14–16]. However, these studies only investigated the benefit of ML in relatively small training datasets, i.e., fewer than 5000 animals, which is smaller than the reference population sizes typically used in current practical applications of GP. In a study in broiler chicken, it was shown that MLP only had a higher accuracy than Bayesian models when the reference population size was smaller than 2100 animals, and the benefit disappeared when the reference population size was larger [17]. In another study where data from ~ 80,000 humans was used for training, there was no benefit of using MLP over BayesB [18]. Although these studies suggest that the benefit of ML models over linear methods decreases when the reference population size increases, the existing evidence remains limited. Therefore, there is a need to investigate the effect of training data size on the benefit of ML models over linear models in a structured manner.

Empirical studies on ML applied in GP used the correlation between predicted values and phenotypes (or the mean squared error (MSE)) as measures of predictive accuracy, because true breeding values (TBV) are unknown in empirical datasets. Considering that breeding values only give rise to additive genetic variance, this approach can overestimate the benefit of ML over linear models for the prediction of breeding values, because ML models may capture non-additive genetic variation, while linear models do not. To overcome this, the benefit of ML models for genetic evaluation could be determined by evaluating the correlation between predictions and highly accurate estimated breeding values, for example from proven sires, in empirical data. If such breeding values are not available, computer simulations of genotypes, breeding values and phenotypes provide a good alternative.

The performance of RF tends to deteriorate with increasing feature numbers [19, 20]. Our previous research found that the prediction accuracy of RF decreases with an increasing number of informative features, i.e. QTL included in the genotype dataset, while the number and proportion of noise features, i.e. non-causal SNPs, had negligible impact on the accuracy [21]. Similarly, some simulation studies found that the prediction accuracies of MLP, convolutional neural network, Bayesian neural networks and RF decreased with an increasing number of QTL, whose genotypes were included as features in the training dataset [15, 22, 23]. In addition, Abdollahi-Arpanahi et al. [22] also reported a similar pattern in scenarios where QTL genotypes were excluded. Conversely, Naderi et al. [24] reported the opposite trend for RF, with prediction accuracy increasing as the number of QTL increased, and the extent of this increase ranged from minor to substantial across scenarios. In that study, however, the QTL genotypes were not included as features in the training dataset, and breeding values were estimated based on a SNP dataset, because QTL are generally unknown. In addition, that study only considered two levels of QTL number, 290 and 725, which are higher than the QTL range (20 to 100) for which we previously observed a dramatic decrease in prediction accuracy [21]. Given these contrasting results, it is still unknown how the number of QTL underlying a trait affects the prediction accuracy of ML models, especially when the number of QTL is less than 100.

The aim of this study is to compare the performance of three different ML models to GBLUP for different data characteristics, including heritability, reference population size, and the number of QTL underlying the trait. Our approach was based on simulations of a livestock population. We compared the prediction accuracy and dispersion bias of GBLUP, random forest (RF), support vector regression (SVR) and multilayer perceptron (MLP).

## Methods

### Simulation

**Fig. 1:**
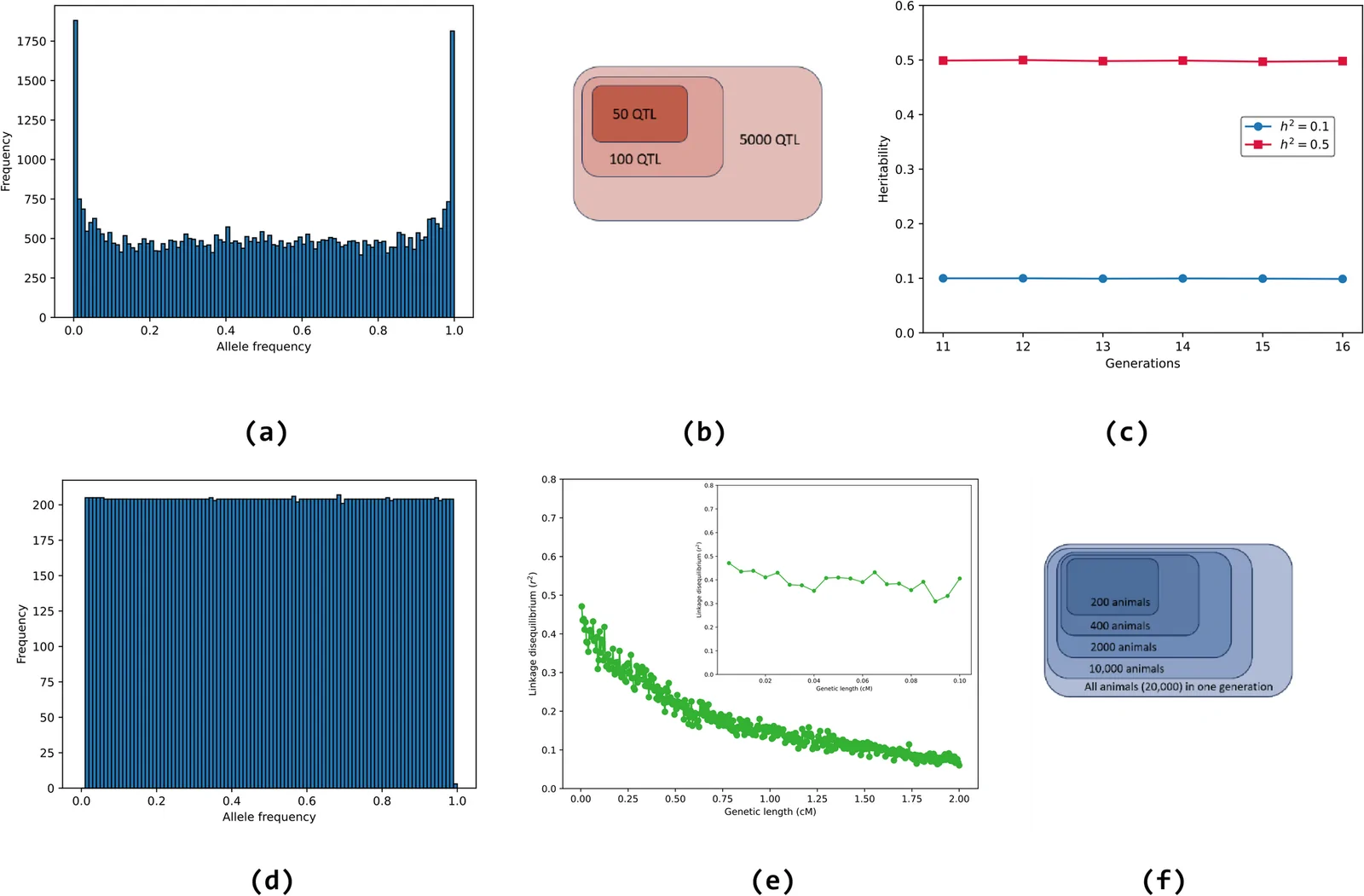
Simulation settings and outputs. **a** histogram of the allele frequency of 80,000 simulated loci (excluding fixed loci) in generation 16 in one replicate, **b** nested QTL effect and location simulation strategy, **c** heritability of target trait for generation 11 to 16 across replicates when there were 50 QTL underlying the trait, **d** histogram of the allele frequency of 20,000 selected SNPs in generation 16 in one replicate, **e** linkage disequilibrium (LD) pattern of generation 16 in one replicate, **f** reference population selection strategy of one generation in generation 11 to 15 for different reference population size

The genotype dataset was simulated with QMSim [25], using the parameter file in Additional file 1. The simulation process mimicked a historic pig population in terms of the linkage disequilibrium (LD) pattern. In the first generation of the historical population, 3000 animals with equal numbers of both sexes were simulated. The population size gradually decreased to 200 in generation 1400, and was maintained at this level until generation 1600 to generate LD. After that, the population size linearly increased to 10,200 in generation 1605 to obtain the required number of individuals. We simulated 10 pairs of autosomes, each 100 cM in length, with 80,000 loci randomly distributed across the genome. After the historical population, 16 generations (numbered as 1–16) were simulated, using an equal sex ratio and litter size of 2. In each generation, we selected and randomly mated the 200 males with the highest EBVs to all 10,000 females to become parents of the next generation. The EBV were estimated for a single trait, referred to as the selection trait, calculated by QMSim with an accuracy of 0.8. Because selection leads to a reduction of heritability for the selection trait [26], we additionally simulated a trait, referred to as the target trait, for the purpose of comparing prediction accuracies across scenarios (details are provided later). The target trait was only simulated for animals from generation 11 to 16. The distribution of allele frequencies of the segregating loci was U-shaped in generation 16 (Fig. 1a).

To explore the impact of different numbers of QTL underlying the target trait, we simulated three trait architectures affected by either 50, 100, or 5000 QTL. These represent traits with a very small (50) and small number of QTL (100), while the 5000 QTL trait represents a polygenic trait. We first randomly sampled 5000 from the 80,000 available loci to serve as QTL. For each QTL, we sampled an additive QTL effect from a standard normal distribution with a mean of 0 and a standard deviation of 1. Given that QTL were randomly sampled, the QTL allele frequency distribution for the 5000 QTL was U-shaped from generation 11 to 16 (Additional file 2: Fig. S1). From the total set of 5000 QTL and their corresponding effects, we randomly selected 100 QTL, and then further selected 50 QTL from within the 100 QTL. Thus, the smaller QTL sets were always subsets of the larger ones (Fig. 1b). For generations 11 to 16, true breeding values (TBVs) of all animals were calculated by multiplying the centered QTL genotypes with their corresponding simulated QTL effects.

We used two levels of trait heritability for the target trait, 0.1 and 0.5. Based on the given heritability and the simulated variance of TBV, representing the additive genetic variance, we calculated the corresponding residual variance. The residual effects were then sampled from a normal distribution with the calculated residual variance and a mean of 0, and phenotypes were obtained by summing the breeding values and the residual effects. In this way, the resulting heritability of the target trait was close to the set value in all generations (Fig. 1c, and Additional file 2: Fig. S2) and across scenarios. Additionally, 20,000 segregating loci that were not sampled as QTL were selected as SNPs for GP, by sampling a specified number of markers across predefined allele frequency intervals to ensure a uniform allele frequency distribution in generation 16 (Fig. 1d). As intended, the obtained LD pattern of generation 16 was comparable to the LD pattern observed in pig populations (Fig. 1e) [27].

The final dataset consisted of the genotypes and phenotypes for the target trait of all 120,000 animals from generations 11 to 16. Animals from generations 11 to 15 were used as the reference population to predict breeding values for 500 selection candidates that were randomly selected from animals in generation 16. We used four reference population size levels: 1000, 2000, 10,000, and 50,000. For each of the reference population sizes, animals were randomly selected without considering sex, and sampled animals were equally distributed across generations 11–15. For example, with 10,000 animals in the reference population, 2000 animals were sampled from each of the 5 generations. Note that the smaller reference populations were subsets of the larger ones in a nested structure (Fig. 1f). In total, we evaluated 24 scenarios based on all combinations of simulation parameters (QTL number (3) × heritability (2) × reference population size (4) = 24 scenarios). We generated 21 simulation replicates, of which one was reserved for hyperparameter tuning of the ML models, and the other 20 were used for analysis. For each scenario, we analyzed the same set of 20 simulation replicates with all considered models.

### Models

#### GBLUP

The genomic best linear unbiased prediction (GBLUP) model applied in this study is expressed as:

$$\:\mathbf{y}=\mathbf{1}\mu\:+\mathbf{Z}\mathbf{u}+\mathbf{e}\:,$$

where $\:\mathbf{y}$ is the vector of phenotypic values, *µ* is the overall population mean, $\mathbf{1}$ is a vector of ones. The vector $\:\mathbf{u}$ represents the genomic estimated breeding values (GEBVs) of animals following a normal distribution *N*($\mathbf{0}$, $\:\mathbf{G}{\sigma\:}_{u}^{2}$), where $\:{\sigma\:}_{u}^{2}$ is the additive genetic variance. The incidence matrix $\:\mathbf{Z}$ links $\:\mathbf{u}$ to $\:\mathbf{y}$, and $\:\mathbf{e}$ is the vector of random errors, following a normal distribution of *N*($\mathbf{0}$, $\:\mathbf{I}{\sigma\:}_{e}^{2}$ ), where $\:\mathbf{I}$ is the identity matrix, and $\:{\sigma\:}_{e}^{2}$ is the residual variance. The genomic relationship matrix $\:\mathbf{G}$ was computed following VanRaden [6]:

$$\mathbf{G} = \frac{\mathbf{W}\mathbf{W}'}{2\sum_j p_j (1-p_j)}$$

where $\:\mathbf{W}$ is the matrix of centered SNP genotypes with elements *w*_*ij*_ = *x*_*ij*_ *− 2p*_*j*_, where *x*_*ij*_ is an element of matrix $\:\mathbf{X}$ containing the genotype for animal *i* at locus *j* coded as 0, 1, or 2, and *p*_*j*_ is the frequency of the allele for which the homozygous genotype at locus *j* is coded as 2.

#### Random forest

Random forest (RF), a ML algorithm introduced by Breiman [11], builds an ensemble of decision trees, each trained on a slightly different subset of features. The final prediction is derived by averaging the outputs of all trees. The RF model can be formally written as:

$$\:\mathbf{y}=\sum\:_{\mathrm{t}=1}^{\mathrm{T}}{\mathbf{h}}_{\mathbf{t}}\left(\mathbf{y};{\mathbf{F}}_{\mathrm{m},\mathrm{t}}\right)\:,$$

where $\:\mathbf{y}$ is the vector of phenotypes, $\:{\mathbf{h}}_{\mathbf{t}}\left(\mathbf{y};{\mathbf{F}}_{\mathrm{m},\mathrm{t}}\right)$ denotes the regression function of the *t*-th decision tree, constructed using a specific subset of * m* features (i.e. SNPs), drawn from the matrix of genotypes $\:\mathbf{F}$. Each tree constructs a decision path by splitting the data at various feature thresholds, ultimately leading to a terminal (leaf) node that corresponds to a predicted value. We tuned RF for four hyperparameters, namely n_estimators (number of trees in the forest), max_depth (maximum depth of the tree), min_samples_leaf (minimum number of samples required to be at a leaf node), and max_features (number of features to consider when looking for the best split).

#### Support vector regression

Support vector regression (SVR) is a supervised learning method derived from support vector machines, designed to model the relationship between input features and the dependent variable [28]. The detailed methodology and mathematical formulations of SVR are provided in Additional File 3. SVR uses a linear or nonlinear kernel function to map the input space (SNPs) to a higher dimensional feature space, and performs modelling and prediction on the feature space. In this study, we used a radial basis function (RBF) kernel, a polynomial kernel, or a linear kernel. The SVR model was tuned by performing a grid search over the following hyperparameters: the regularization constant C, the epsilon margin ε, and the kernel parameter $\:\gamma\:$ for the RBF and polynomial kernels, whereas the linear kernel does not require $\:\gamma\:$.

#### Multilayer perceptron-residual network

**Fig. 2:**
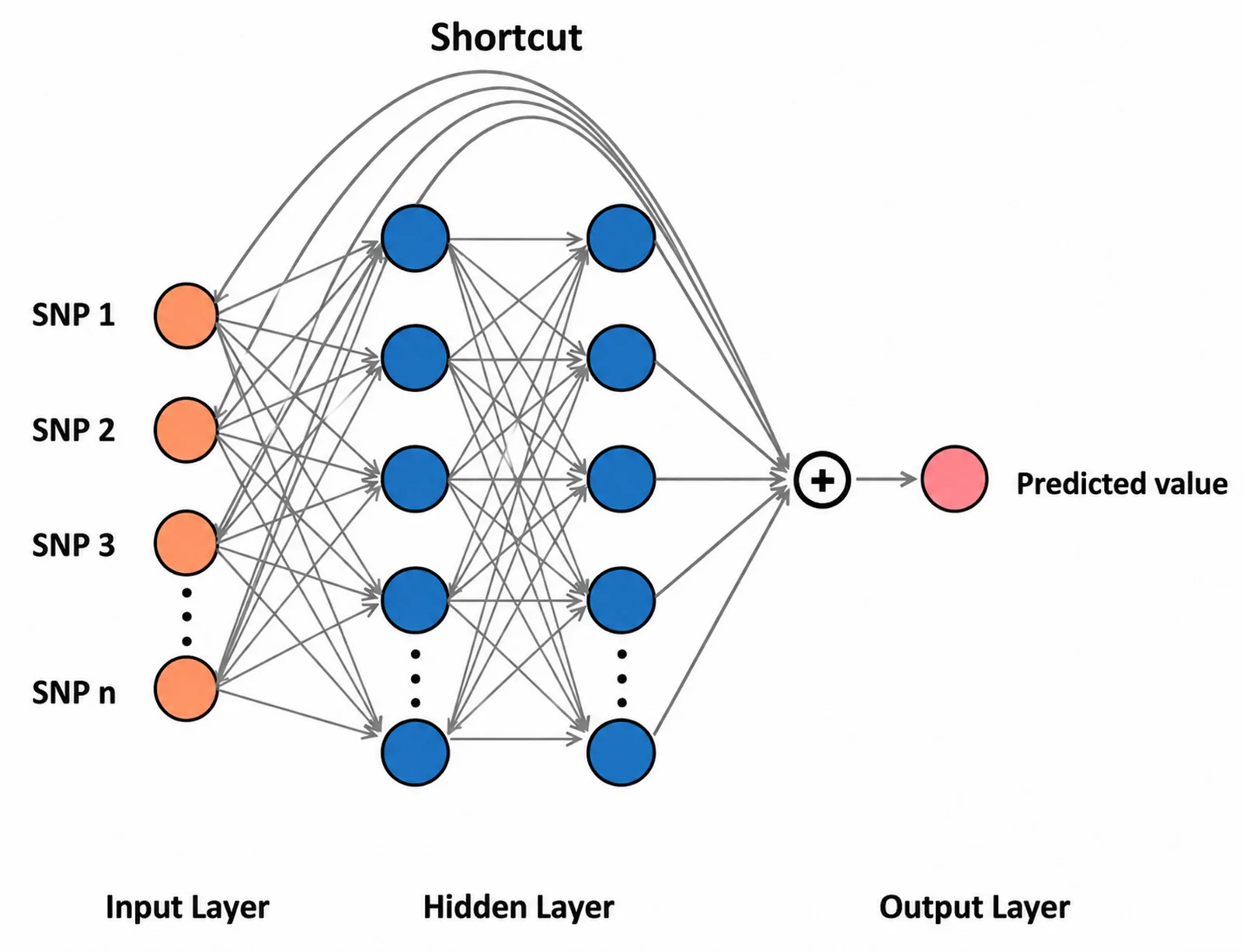
Illustration of MLP-ResNet

Multilayer perceptron (MLP) is a class of feedforward artificial neural networks that consists of an input layer, one or more hidden layers, and an output layer [13]. Each layer consists of a set of neurons, and all neurons are fully connected to the neurons in adjacent layers. The input layer receives the feature matrix (i.e., SNP genotypes), which is then processed through the hidden layers via a series of linear transformations and nonlinear activation functions, and the final output layer produces predicted values (Fig. 2).

We initially chose an ordinary MLP model to maintain architectural simplicity. However, in our preliminary analyses, we observed that this model often suffered from collapse, which is a degenerate state in which the model produced identical predicted values for all validation animals, regardless of their genotype. This phenomenon of output collapse, where the network converges to constant or trivial predictions, is a well-known issue in MLP [29, 30]. To address this issue, we adopted the residual network (ResNet) architecture introduced by He et al. [31], which has been proven to be effective in preventing collapse. ResNet introduces shortcut connections that bypass layers by adding the input layer directly to its output. In our model, hereafter called MLP-ResNet (Fig. 2), we incorporated shortcut connections into the MLP architecture by adding residual links between input and output of certain hidden layers. The MLP-ResNet model was tuned by performing a grid search over the following hyperparameters: the activation function, the number of neurons in each hidden layer, the number of residual blocks, regularization parameter L2 and learning rate.

#### Hyperparameter tuning for machine learning models

We used the dataset from one of the 21 replicates for tuning the hyperparameters of ML models. For each of the 24 scenarios, hyperparameters were tuned independently. To mimic a realistic setting where available reference data needs to be split in training and validation for hyperparameter tuning, we used animals from generation 11 to 14 for training, while using animals from generation 15 for validation. For example, for the reference population of 1000 animals, we used 800 animals from generation 11 to 14 for training and 200 animals from generation 15 for validation.

For the RF model, we applied a grid search to tune the hyperparameters. The values tested were: max_features = [500, 1000, 2000, 5000], max_depth = [5, 8, 10, 20, 30, None], and min_samples_leaf = [1, 3, 10]. For this tuning process, the number of trees (n_estimators) was initially fixed at 500. Additionally, for the scenario with the largest reference population size, we further evaluated the prediction accuracy using n_estimators values of 1500 and 3000 to assess the trade-off between computational cost and prediction accuracy. Since larger n_estimators generally leads to higher prediction accuracy but also substantially increases computation cost, this trade-off needed to be considered carefully. Among all scenarios, the computation cost was highest for the largest reference population size, making it the most critical case for such evaluation.

For the SVR model, we also used a full grid search to optimize the hyperparameters. Three kernel functions were evaluated: linear, radial basis function (RBF), and polynomial. The penalty parameter (C) was tested at values of 0.1, 1, and 10, while the ε-insensitive loss parameter (epsilon) was tested at 0.01, 0.1, and 0.5. For the RBF and polynomial kernels, the kernel coefficient (gamma) was set to “scale”, 0.001, and 0.01.

For the MLP-ResNet model, a full grid search was used to optimize hyperparameters. Three activation functions were evaluated: ReLU, GELU, and ELU. The hidden dimension, which is the number of neurons in each layer, was tested at values of 32, 64, and 128, while the number of residual blocks was varied between 1, 3, and 5. To explore regularization effects, the L2 penalty was tested at 0.0001, 0.001, and 0.01. Additionally, learning rates of 0.0001, 0.001, and 0.005 were assessed to identify the optimal training dynamics.

For each model and each scenario, we chose the optimal hyperparameter settings by picking the combination of hyperparameters that resulted in the highest accuracy in the validation set, measured as the correlation between predicted values and simulated breeding values. The time limit for testing each combination was 2 days, which means that combinations that resulted in a computation time of more than 2 days were not considered for further analysis. The optimal hyperparameters from the tuning replicate were used in the analysis of the other 20 replicates.

### Prediction evaluation and computational implementation

For each model and each scenario, accuracy and dispersion bias of predictions were evaluated in the test set containing 500 animals from generation 16. Accuracy was computed as the correlation coefficient between predicted value and TBV, and dispersion bias was computed as the deviation of the regression coefficient of TBV on predicted value from the expected value of 1.

For GBLUP, the genomic relationship matrices were computed using the calc_grm software [32], and variance component estimation and genomic estimated breeding values (GEBVs) were computed using the MTG2 software [33]. If the additive genetic variance estimated by MTG2 was negative, prediction accuracy was set to zero, and the corresponding replicate was excluded from the calculation of dispersion bias. For the RF and SVR models, implementations from the Scikit-learn package [34] were used. The MLP-ResNet model was implemented using the Keras deep learning library [35].

## Results

### Tuning hyperparameters

We performed hyperparameter tuning for RF, SVR, and MLP-ResNet using grid search, selecting the best-performing combination for each scenario based on prediction accuracy in the validation dataset in an independent replicate (Additional file 4 to Additional file 6). For all models, the optimal set of hyperparameters varied across different number of QTL underlying the trait, heritabilities and reference population sizes, underscoring the necessity of scenario-specific hyperparameter tuning. For RF and a reference population size of 50,000, the prediction accuracies were 0.784, 0.794, and 0.799 for n_estimators = 500, 1500, and 3000, respectively, while the corresponding computation times were 13, 35, and 60 min. Considering the trade-off between computational cost and prediction performance, we used n_estimators = 1500 for further analyses in RF. With a fixed number of trees, max_features showed the greatest influence on prediction accuracy, while max_depth and min_samples_leaf had a smaller influence. For SVR, analysis using a linear kernel did not converge within two days, even when reference population size was only 1000. Therefore, the linear kernel was excluded from further analyses. When using the RBF kernel, gamma was the most influential hyperparameter, whereas C and epsilon had smaller influence. The polynomial kernel showed robustness to all three hyperparameters, and generally achieved higher accuracy than RBF, therefore the final selected hyperparameter combinations were restricted to those using the polynomial kernel. In MLP-ResNet, all tested hyperparameters, including the activation function, hidden layer dimension, number of residual blocks, L2 regularization, and learning rate, had a substantial impact on prediction accuracy.

### Prediction accuracy

**Fig. 3:**
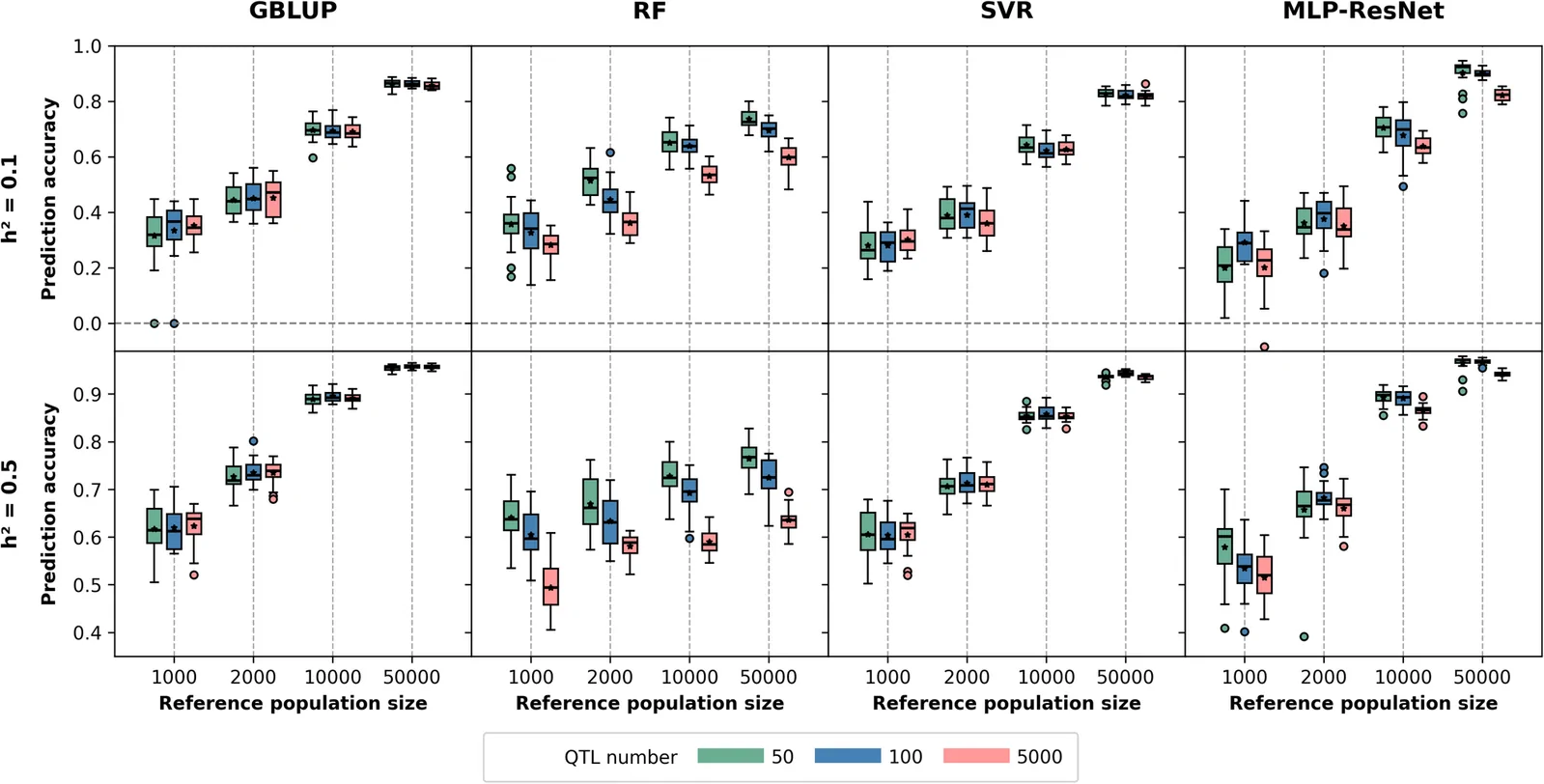
Prediction accuracy of GBLUP, RF, SVR and MLP-ResNet at two heritability levels. Each box plot summarizes the distribution of prediction accuracies across 20 replicates. The boxes represent the interquartile range (IQR), with the median shown as a horizontal line and the average indicated by an asterisk (*). Whiskers extend to the most extreme data points within 1.5×IQR, and data points beyond this range are plotted as outliers

The impact of reference population size, number of QTL, heritability and prediction model on accuracy is provided in Fig. 3. As expected, increasing the size of the reference population consistently improved prediction accuracy across all models and scenarios. GBLUP and SVR showed relatively similar patterns of accuracies across different QTL numbers, while RF and MLP-ResNet showed greater sensitivity to the number of QTL. The accuracy of RF dropped dramatically as the number of QTL increased, with an average difference in accuracy of 0.13 between traits with 50 QTL and 5000 QTL. For MLP-ResNet, the trend in prediction accuracy across number of QTL varied across different reference population sizes. There was generally no clear pattern for the number of QTL when the reference population size was 1000 or 2000. However, when heritability was 0.5 and the reference population size was 1000, prediction accuracy decreased as the number of QTL increased. Prediction accuracy consistently decreased with increasing numbers of QTL when the reference population size was 10,000 or 50,000. For MLP-ResNet, the average difference in accuracy between the traits with 50 and 5000 QTL was 0.06 across reference population sizes 10,000 and 50,000.

### Benefit of ML models in terms of prediction accuracy

**Fig. 4:**
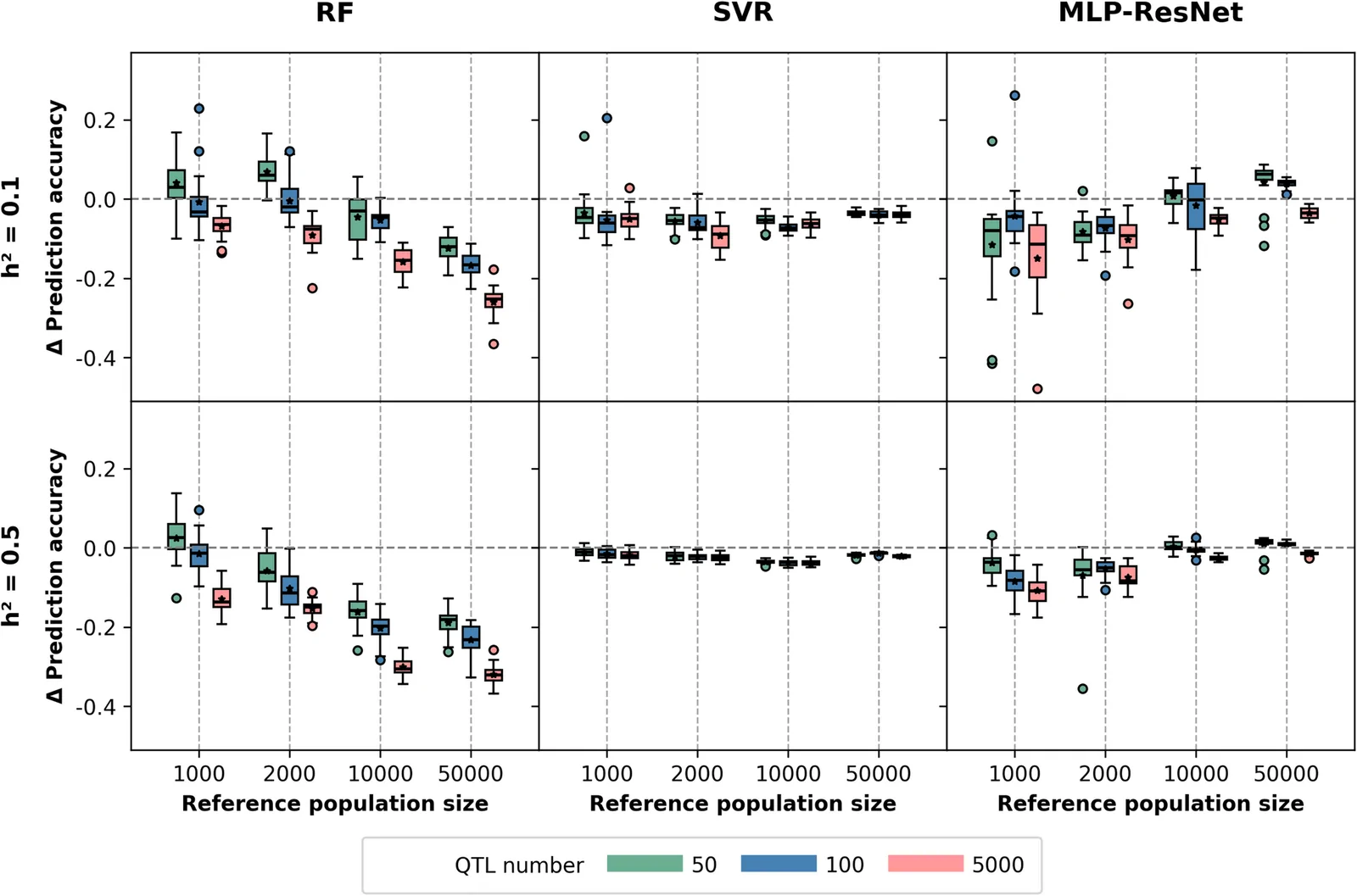
Difference in prediction accuracy between ML models and GBLUP. Box plots are shown as described in the caption of Fig. 3

The benefit of ML models over GBLUP varied across models and was influenced by both reference population size and the number of QTL underlying the trait (Fig. 4). RF outperformed GBLUP only when the number of QTL was 50, particularly when the reference population size was small. This benefit was more pronounced with a low heritability than with a high heritability. The accuracy of SVR was slightly but consistently lower than that of GBLUP across all scenarios. MLP-ResNet showed benefits over GBLUP when the reference population size was at least 10,000 and QTL number was 50 or 100.

### Dispersion bias

**Fig. 5:**
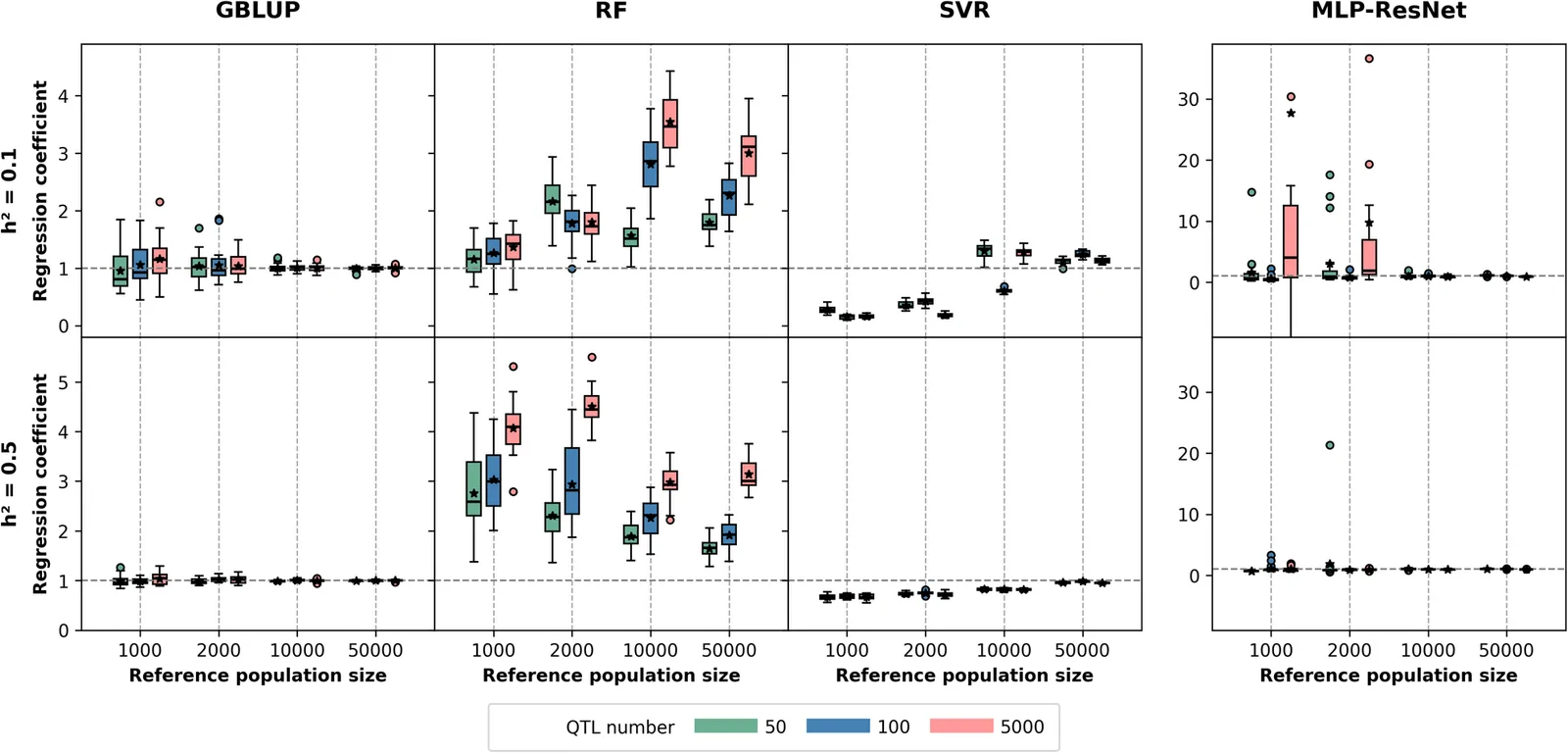
Dispersion bias of GBLUP, RF, SVR and MLP-ResNet at two heritability levels. Box plots are shown as described in the caption of Fig. 3

Dispersion bias varied among prediction models and was influenced by the reference population size, the heritability, and the number of QTL (Fig. 5). For GBLUP, the average dispersion bias was limited, as the coefficient of regression of TBV on the predicted value remained close to 1 across all scenarios, particularly when the reference population size was larger than 2000, while the variation in dispersion bias for small reference populations was larger compared to that for large ones. RF showed under-dispersion across all scenarios. With high heritability, the degree of bias generally decreased as the reference population size increased, while with low heritability, this pattern was less clear. With a low heritability and a reference population size of 2000, the degree of bias decreased as the number of QTL increased, while in all other scenarios, the degree of bias increased as the number of QTL increased. Overall, SVR showed relatively low dispersion bias, but higher than GBLUP, especially when heritability was high. MLP-ResNet showed the greatest instability in dispersion bias with low heritability, with extreme values (regression coefficient > 10) occurring with small reference population sizes. With high heritability, the bias was substantially reduced and the regression coefficient remained close to 1 when the reference population size was larger than 10,000.

## Discussion

We aimed to compare the GP performance of RF, SVR, and MLP-ResNet to GBLUP for different QTL numbers underlying the trait, heritabilities and reference population sizes. Our results showed that the number of QTL underlying the trait is a key factor that affects the prediction accuracy of RF and MLP-ResNet. Generally, the prediction accuracy decreased as the QTL number increased. In addition, we found that RF outperformed GBLUP in small reference populations combined with a low QTL number, while MLP-ResNet outperformed GBLUP only in large reference populations with a low QTL number. In all other scenarios, GBLUP outperformed the three ML models in terms of prediction accuracy and dispersion bias.

To compare the computation time of different models using 1 CPU, we measured computation time for all models, using 1 replicate, 2 level of heritability, 3 levels of QTL number, while fixed the reference population size to 50,000. MLP-ResNet was the fastest model, with computation times ranging from 16 to 80 min (Additional file 7: Table S1), followed by RF, which required 82 to 520 min, and GBLUP, which required 299 to 645 min. SVR required the longest computation time, ranging from 584 to 6572 min. Different combinations of hyperparameters of SVR results in different computation time, and heritability seemed to have a large impact on computation time (Additional file 7: Table S2). Note that the computation times for ML models do not include time required for hyperparameter tuning, while the computation time for GBLUP did include time required for variance component estimation. Although heritability is typically provided as an input in practical genetic evaluation, ML models do not require such input. Therefore, for a fair comparison across models, we performed variance component estimation for GBLUP, which increased its computation time.

### The influence of the QTL number

In general, the accuracy of GP is expected to increase when the number of QTL decreases, because when the number of QTL is lower, each QTL explains a larger proportion of genetic variance [36]. GBLUP is, however, known to result in more or less the same prediction accuracy for different QTL numbers [15, 22–24, 36, 37], which is in agreement with our findings. The reason for this is that GBLUP is based on the infinitesimal model, which assumes that many genes with small effects control the trait. Therefore, GBLUP is not optimized for traits controlled by a small number of QTL. This is in contrast to for example Bayesian variable selection models, which are better suited for traits with a small number of QTL [36, 37]. We also observed that SVR was not affected by QTL number across heritabilities, which generally aligns with results from other studies [23].

In contrast to GBLUP and SVR, prediction accuracy of RF indeed increased when the number of QTL decreased, across all reference population sizes and heritabilities. This result is consistent with our previous study, although in that study QTL genotypes were explicitly included in the genotype dataset [21]. Although the increase of QTL number also results in the increase of the ratio of QTL to SNPs, our previous study found that this ratio was not responsible for the decrease in prediction accuracy. Both Ghafouri-Kesbi et al. [23] and Abdollahi-Arpanahi et al. [22] reported a similar pattern for RF when QTL genotypes were explicitly included in the dataset. Notably, Ghafouri-Kesbi et al. [23] also examined a scenario excluding QTL genotypes and reported a higher accuracy with 100 QTL than with 1000 QTL. In contrast, Naderi et al. [24] simulated traits with either 290 or 750 QTL underlying the trait and found that RF accuracy increased as the QTL number increased. However, they did not report how the RF hyperparameters were tuned, so it remains uncertain whether the hyperparameters were adjusted to each scenario.

We attribute our observed decrease in prediction accuracy of RF with increasing QTL number to the way RF partitions features through binary splits. When the number of QTL is small, each QTL contributes a larger effect and explains a larger proportion of the genetic variance. This increases the probability of selecting highly informative features (top-SNPs) at early splits. Because the first few splits determine the major structure of the decision trees, placing strong-effect features at these splits enables the model to partition the data in a way that reflects the true genotype–phenotype relationships, thereby improving prediction accuracy. As the number of QTL increases, effects of QTL are diluted and highly informative features become harder to identify at early splits. As a result, a greater proportion of splits rely on weak or non-informative features, which ultimately results in a lower prediction accuracy.

MLP-ResNet showed a similar sensitivity to QTL number as RF, where prediction accuracy generally decreased as the QTL number increased, which is consistent with results from other studies using neural networks [15, 22]. Unlike tree-based models that perform implicit feature selection via early splits, MLP-ResNet relies on gradient-based optimization to adjust a large number of parameters jointly. When only a few QTL give rise to most of the genetic signal, gradients point more clearly toward informative directions and the network can learn meaningful genotype–phenotype mappings more easily. As QTL number increases, the per-locus effect size and the signal-to-noise ratio per feature decrease, gradients become noisier, and optimization is less likely to converge on stable, generalizable patterns. When the predictive signal is dispersed across many weak features the network must integrate many small signals across layers and parameters, which increases the risk of fitting spurious noise unless sample size and regularization are sufficient. Together, these mechanisms may explain why MLP-ResNet performs better with few, large-effect QTL but worse as QTL number increases.

Taking everything together, our findings highlight that the QTL number impact the accuracy of ML models in GP, in particular for tree-based and neural network models. Therefore, for newly developed GP models, it is essential to evaluate their performance using simulated datasets across different QTL numbers. In empirical datasets, deriving the potential influence of QTL number is much more complicated, because the precise genetic architecture of many traits are unknown.

### Benefit of ML models over GBLUP

RF outperformed GBLUP in terms of the prediction accuracy when both the number of QTL and the reference population size were small, whereas MLP-ResNet outperformed GBLUP when the number of QTL was small and the reference population size was large. In all other scenarios, GBLUP was the model with the highest prediction accuracy. As indicated before, GBLUP is based on the infinitesimal model, which makes it most suitable for traits controlled by a very large number of loci with small effects. When the underlying genetic architecture deviates from this assumption, as in scenarios with a low number of QTL, ML models that are not constrained by this assumption, such as RF and MLP-ResNet, may have an advantage. At the same time, however, ML models are designed to fit and predict phenotypes, making them inherently less suited for direct TBV prediction.

The performance of GBLUP is known to be influenced by population structure and linkage LD, and GBLUP can exploit genetic relationships between individuals, even in the absence of LD [38, 39]. In contrast, ML models might rely more directly on marker-QTL associations driven by LD, instead of relationship information alone. This would suggest that the relative performance of ML models and GBLUP is not only determined by the genetic architecture of the trait, but can also be influenced by the LD pattern and population structure. The influence of LD and population structure on the potential benefit of ML over GBLUP will be investigated in our future study.

Our findings on the benefit of RF over GBLUP are in line with results reported by simulation studies [22–24, 40] (Additional file 8: Table S1). In empirical datasets, a benefit of RF over GBLUP is generally observed in cases with a relatively small reference population and low heritability (Additional file 8: Table S1). However, we found one study that was not in line with our results, which will be discussed in more detail here. In a study on GP for total number of piglets born in Yorkshire pigs, RF outperformed GBLUP with a reference population of 2,053 animals [16]. In contrast, another study on the same trait and breed, but with a smaller reference population (950 animals), reported that the prediction accuracy achieved by RF was lower than that of GBLUP [41]. A possible explanation is that the discrepancy resulted from population-specific factors, such as differences in genetic relationships, inbreeding levels, and selection pressures. These differences, as reflected in different heritability between the two populations (0.12 in the former and 0.20 in the latter), can affect the benefit of RF over GBLUP. Another possible explanation is that the differences between the two studies might also have arisen from differences in implementation of ML models.

Our results showed that MLP-ResNet outperforms GBLUP when the reference population size was large and the number of QTL was low. This is in contrast with earlier observations. Several studies with empirical data reported lower accuracies with MLP compared to GBLUP with large reference populations [18], and higher accuracies with small reference populations [17, 42–44] (Additional file 8: Table S2). For example, Bellot et al. [18] conducted a study with ~ 80,000 human samples and found that MLP achieved lower accuracy than linear models. It should be noted that they used three different sets of hyperparameters across all traits, rather than optimizing them for each trait. According to their results, the performance indeed varied considerably across different hyperparameter settings. In another study, Passafaro et al. [17] analyzed a population of ~ 68,000 broilers and found that MLP only outperformed GBLUP when the reference population size was small (i.e., less than 2032). Although the discrepancies between our results and the studies using empirical data could be explained by the differences in model used (MLP-ResNet versus MLP), the use of MLP for GP seems promising in some situations, and warrants further study.

The results of our study and those of empirical studies might indicate that, with small reference populations, the performance of MLP-ResNet may be very sensitive to stochastic variation and the chosen set of hyperparameters. To study this sensitivity, we first re-analyzed a replicate that initially showed a particular poor performance using different random seeds, and observed substantial differences in prediction accuracy between the random seed values (Additional file 9: Table S1). Second, we applied the same hyperparameter tuning strategy but optimized it specifically for this replicate. The best-tuned model achieved an accuracy close to, but still lower than, that of GBLUP. Notably, dispersion bias was also improved under the optimal parameter setting. In summary, these findings indicate that when the reference population size is small, both stochastic model variation and hyperparameter tuning influenced the performance of MLP in GP. A practical implication is that, especially with small reference populations, it may be useful to try different random seed numbers when irregular prediction accuracies are observed for MLP.

The observed benefits of RF and MLP-ResNet over GBLUP suggest that they can be promising alternatives for GP when the number of QTL underlying the trait is small (i.e. ≤ 100). Although most quantitative traits are highly polygenic, some are affected by a limited number of major genes that explain a large proportion of genetic variance, forming an oligogenic–polygenic mixed structure. Such traits may be treated as being controlled by a small number of QTL. For example, five major QTL explain more than 30% of the phenotypic variation for grain length, grain width, and 1000-grain weight in rice [45, 46]. Similar cases also exist in animals; for example, six genes can explain nearly half of the phenotypic variation in body size of dogs [47]. Likewise, traits such as fat percentage in dairy cattle and total number of piglets born in pigs also show potential for applying ML in GP [48, 49]. In humans, one study detected 75 QTL associated with Alzheimer’s disease, while a single gene explained up to 20% of the phenotypic variance [50]. For such traits, RF and MLP-ResNet are promising GP models. When the reference population is small (i.e., fewer than 2000), RF might outperform GBLUP, whereas when it is large (i.e., greater than 10,000), MLP-ResNet may be more suitable. Moreover, the computation time of MLP-ResNet is substantially shorter than that of GBLUP, which provides another advantage in large-scale datasets.

To further explore model performance under more extreme conditions, we evaluated additional scenarios with very low heritability (0.01) (Additional file 10: Fig. S1 and Fig. S2). This was motivated by two considerations: first, to assess model performance for traits with very low heritability; and second, because our main results suggested that the relative advantage of ML models over GBLUP might increase as heritability decreases. Because SVR did not show benefit over GBLUP in our main results, we did not include SVR in this analysis. Because prediction accuracy for GBLUP was set to zero when the estimated variance component was negative, ML models resulted in a higher number of negative prediction accuracy values at such low heritability. However, we even observe an advantage of RF over GBLUP when the number of QTL was 50 and the reference populations larger than 10,000. These results indeed suggest that the relative benefit of RF over GBLUP increases as heritability decreases (Additional file 11: Fig. S1). MLP-ResNet also showed benefit over GBLUP when the reference population size was smaller than 2000. While MLP-ResNet resulted in very low or even negative prediction accuracies in some replicates when the reference population size was larger than 10,000, this led to larger variation in prediction accuracy compared to GBLUP. To further study this phenomenon, we applied the same hyperparameter tuning strategy but optimized it specifically for one replicate in which the prediction accuracy of MLP-ResNet was 0.027, and the best-tuned hyperparameters combination achieved an accuracy of 0.368 (details not shown). This indicates that the increased variability is likely due to suboptimal hyperparameter settings that require replicate-specific tuning. When the reference population size was 50,000 and the number of QTL was 50, MLP-ResNet resulted in a 0.03% higher accuracy than GBLUP in the presence of outliers. Overall, these additional results confirm that our main conclusions remain valid.

### Performance of SVR

Our results showed that the prediction accuracy of SVR was slightly lower than that of GBLUP across scenarios, which is consistent with our previous study as well as other studies [21, 23, 51, 52]. However, some studies have reported that SVR can achieve higher prediction accuracy than GBLUP in some situations [14, 16, 41]. Notably, in all these cases where SVR outperformed GBLUP, the predicted value was compared to phenotypes and not breeding values, and the reference population size was smaller than 4000. Therefore, this benefit may be a result of SVR’s ability to capture non-additive genetic effects. We therefore do not recommend SVR for GP of breeding values, especially not because the computation time is much longer.

### Hyperparameter tuning

Our results highlight the importance of hyperparameter tuning for the performance of ML models in GP. Based on the results of tuning process, RF appeared relatively robust to changes in parameter settings, whereas SVR and MLP were highly sensitive to hyperparameter changes. Moreover, we found that the reference population size and the number of QTL underlying the trait affected the optimal hyperparameter settings. The optimal hyperparameter combination seems to be dataset-specific, suggesting that breeding companies would need to conduct separate tuning process for different populations, traits, and when reference population largely increases. This requirement makes the practical use of these ML models in livestock breeding challenging.

### Prediction accuracies being higher in test than in training data

We observed that, in some scenarios of RF, the average correlation across replicates between the predicted value and TBV was higher in the test dataset than that in the training dataset (Additional file 12: Table S1), which is counterintuitive. In addition, we noted that when heritability was 0.1, this phenomenon occurred more frequently than when heritability was 0.5. We think that this is because the RF model is trained to predict the phenotype. Therefore, it can inadvertently fit spurious patterns in the residuals of the animals in the training set, particularly when heritability is low (Additional file 13: Fig. S1). Spuriously explaining variation in the residuals in the training set deteriorates the prediction of the breeding values in the training set, because it increases the variance of the predictions, without increasing the covariance with the TBVs. This additional variation in the predictions is however not propagated to the test set, simply because the spurious associations are not transferrable. To test this hypothesis, we re-analyzed a single replicate using TBV as the input, i.e. the residual error in the training set was 0 (heritability = 1) to prevent the model to fit any noise. We indeed observed that in this case, the accuracy in the training set was higher than the accuracy in the test set (Additional file 12: Table S2). This supports our hypothesis that RF fits the residual noise in the training dataset, which can deteriorate the breeding value prediction of the training animals.

### Limitation of this study

This study has several limitations that should be acknowledged. First, we simulated only additive genetic effects and no non-additive genetic effects, because we were interested in the accuracy of predicting additive genetic values (i.e. breeding values). Simulating non-additive genetic effects and focusing on prediction of phenotypes may change the relative performance of GP models, because ML models may be able to capture non-additive genetic effects. Second, we chose to implement basic ML models rather than more advanced ones to enhance the generalizability and interpretability of our results. For example, XGBoost is considered more advanced than RF among ensemble models, and it is possible that more advanced models result in higher prediction accuracy. Third, our simulations assumed a normal distribution of QTL effects which may result in a conservative performance of ML models. Using alternative distributions of QTL effects, such as gamma distribution, may further increase the benefit of RF when both the reference population size and the number of QTL are small. This is because a gamma distribution concentrates a larger proportion of the genetic variance to a few QTL, which is functionally similar to reducing the number of QTL. Finally, because hyperparameters were not tuned for each replicate individually, the prediction accuracies reported here represent conservative estimates and may not reflect the maximum achievable accuracy with ML models.

## Conclusions

This study provides a comprehensive comparison of ML methods and GBLUP for GP for different QTL numbers underlying the trait, reference population sizes and heritabilities. Our results demonstrated that the benefit of ML models over GBLUP depends on the number of QTL and the size of the reference population. Specifically, RF outperformed GBLUP when both the number of QTL and the reference population size were small, whereas MLP-ResNet showed an advantage when the number of QTL was small and the reference population was large. In all other situations, GBLUP outperformed the ML models. These findings indicate that a benefit of ML models over GBLUP may be expected in scenarios where the infinitesimal model assumption of GBLUP does not hold.

From a practical perspective, our results carry important implications for the application of ML models for GP in animal and plant breeding, but also in polygenic risk score prediction for human diseases. Our results emphasize that when introducing a new GP model, particularly tree-based or neural network methods, it is essential to evaluate its performance on simulated datasets across different numbers of QTL and reference population sizes, because the benefit might strongly vary across those settings. In practice, ML models may be considered as alternatives to GBLUP for traits controlled by a relatively small number of QTL (e.g., ≤ 100). In this case, RF appears advantageous in scenarios with small reference populations, while MLP-ResNet shows better performance when the reference population size is large. Traits with oligogenic–polygenic mixed architectures may be promising target traits for ML models in GP.

## Supplementary Information

Below is the link to the electronic supplementary material.

Supplementary Material 1

Supplementary Material 2

Supplementary Material 3

Supplementary Material 4

Supplementary Material 5

Supplementary Material 6

Supplementary Material 7

Supplementary Material 8

Supplementary Material 9

Supplementary Material 10

Supplementary Material 11

Supplementary Material 12

Supplementary Material 13

## Data Availability

QMSim input file used to generate the data during this study is Additional file 1. Seeds for QMSim simulation and codes for analysis can be found via https://git.wageningenur.nl/yang110/.
