# Supplementary material for: Impact of QTL number, heritability and reference population size on the benefit of machine learning models over GBLUP in genomic prediction
Source: Genet Sel Evol. 2026 Jul 13;58:49. doi: 10.1186/s12711-026-01065-6 (PMC13491627; doi:10.1186/s12711-026-01065-6)
Supplement: Supplementary file 1 [file 12711_2026_1065_MOESM8_ESM.pdf]

Additional file 8:

Table S1. Summary of the GP study compared RF with GBLUP

Species (Reference)		Training size	h^2	Trait	QTL number	Benefit of RF
Animal						
	Dairy cattle [1]	11790	0.3	Sire conception rate	N/A	No
	Dairy cattle [1]	11790	0.3	N/A*	100/1000	No
	Cattle [2]	16000	0.1/0.3	N/A	290	No
	N/A [3]	1000	0.1	N/A	100/1000	Yes/No
	N/A [3]	1000	0.3/0.5	N/A	100/1000	No
	Pig [4]	2053	0.12	Piglets born	N/A	Yes
	Pig [5]	950	0.2	Piglets born	N/A	No
	Banana shrimp [6]	562	0.25-0.60	Six different traits	N/A	Yes
	Hanwoo Cattle [7]	7234	0.35-0.44	Four different traits	N/A	No
Plant						
	Sugarcane [8]	2912	0.72/0.79 /0.89	TCH/CCS/Fiber	N/A	Only for TCH
	Rice [9]	985	0.31	Resistance to planthopper	N/A	Yes
	N/A [10]	< 3000	0.7	N/A	5	Yes
	Sugarcane [11]	4702/1988	0.36/0.34	Smut/Pachymetra root rot	N/A	Yes/No

* Simulated data

Table S2. Summary of the GP study compared MLP with linear models

Species (Reference)	Training size	h^2	Trait	QTL number	Linear model	Benefit of MLP
Animal						
Dairy cattle [1]	11790	0.3	Sire conception rate	N/A	GBLUP	No
Dairy cattle [1]	11790	0.3	N/A*	100/1000	GBLUP	No
Pig [5]	1187-3916	0.07-0.62	Different traits in 5 populations	N/A	GBLUP	No**
Catfish [12]	5328	~0.45	Resistance to disease	N/A	GBLUP	Yes
Broiler [13]	678-67741	0.23	Body weight	N/A	BayesCπ	No***
Human [14]	71529/80000	0.32/0.67	Bone heel mineral Density/Height	N/A	BayesB	No
Beef [15]	3079	Not mentioned	Marbling score	N/A	BayesCπ	Yes
Plant						
Sugarcane [8]	2912	0.72/0.79/0.89	TCH/CCS/Fiber	N/A	GBLUP	Only for TCH
Rice [9]	985	0.31	Resistance to planthopper	N/A	GBLUP	No
Sugarcane [11]	4702/1988	0.36/0.34	Smut/Pachymetra root rot	N/A	GBLUP	No/Yes
Maize, rice… [16]	391-5014	Not mentioned	Different traits in 6 species	N/A	rrBLUP	No
Wheat [17]	306	~0.85/~0.97	Grain yield/Days to heading	N/A	BayesA	Yes

* Simulated data, ** 4 out of 16 different comparison among different population and traits showed benefit, *** When reference population size is 678 and 2032, MLP showed benefit

Reference

1. Abdollahi-Arpanahi R, Gianola D, Penagaricano F. Deep learning versus parametric and ensemble methods for genomic prediction of complex phenotypes. Genet Sel Evol. 2020;52:12.
2. Naderi S, Yin T, König S. Random forest estimation of genomic breeding values for disease susceptibility over different disease incidences and genomic architectures in simulated cow calibration groups. Journal of Dairy Science. 2016;99:7261-73.
3. Ghafouri-Kesbi F, Rahimi-Mianji G, Honarvar M, Nejati-Javaremi A. Predictive ability of Random Forests, Boosting, Support Vector Machines and Genomic Best Linear Unbiased Prediction in different scenarios of genomic evaluation. Anim Prod Sci. 2017;57:229-36.
4. Wang X, Shi S, Wang G, Luo W, Wei X, Qiu A, et al. Using machine learning to improve the accuracy of genomic prediction of reproduction traits in pigs. J Anim Sci Biotechnol. 2022;13:60.
5. Su RL, Lv JB, Xue YH, Jiang S, Zhou L, Jiang L, et al. Genomic selection in pig breeding: comparative analysis of machine learning algorithms. Genetics Selection Evolution. 2025;57.
6. Nguyen NH, Vu NT, Patil SS, Sandhu KS. Multivariate genomic prediction for commercial traits of economic importance in Banana shrimp. Aquaculture. 2022;555.
7. Srivastava S, Lopez BI, Kumar H, Jang M, Chai HH, Park W, et al. Prediction of Hanwoo Cattle Phenotypes from Genotypes Using Machine Learning Methods. Animals-Basel. 2021;11.
8. Chen CS, Powell O, Dinglasan E, Ross EM, Yadav S, Wei XM, et al. Genomic prediction with machine learning in sugarcane, a complex highly polyploid clonally propagated crop with substantial non-additive variation for key traits. Plant Genome-Us. 2023;16.
9. Zhou C, Jiang WH, Guo JP, Zhu LL, Liu LJ, Liu SY, et al. Genome-wide association study and genomic prediction for resistance to brown planthopper in rice. Frontiers in Plant Science. 2024;15.

10. Farooq M, van Dijk ADJ, Nijveen H, Mansoor S, de Ridder D. Genomic prediction in plants: opportunities for ensemble machine learning based approaches. F1000Res. 2022;11:802.
11. Chen CS, Bhuiyan SA, Ross E, Powell O, Dinglasan E, Wei XM, et al. Genomic prediction for sugarcane diseases including hybrid Bayesian-machine learning approaches. Frontiers in Plant Science. 2024;15.
12. Vu NT, Phuc TH, Oanh KTP, Sang NV, Trang TT, Nguyen NH. Accuracies of genomic predictions for disease resistance of striped catfish to using artificial intelligence algorithms. G3-Genes Genom Genet. 2022;12.

13. Passafaro TL, Lopes FB, Dorea JRR, Craven M, Breen V, Hawken RJ, et al. Would large dataset sample size unveil the potential of deep neural networks for improved genome-enabled prediction of complex traits? The case for body weight in broilers. BMC Genomics. 2020;21:771.
14. Bellot P, de Los Campos G, Perez-Enciso M. Can Deep Learning Improve Genomic Prediction of Complex Human Traits? Genetics. 2018;210:809-19.
15. Okut H, Wu XL, Rosa GJ, Bauck S, Woodward BW, Schnabel RD, et al. Predicting expected progeny difference for marbling score in Angus cattle using artificial neural networks and Bayesian regression models. Genet Sel Evol. 2013;45:34.
16. Azodi CB, Bolger E, McCarren A, Roantree M, de Los Campos G, Shiu SH. Benchmarking Parametric and Machine Learning Models for Genomic Prediction of Complex Traits. G3 (Bethesda). 2019;9:3691-702.
17. Perez-Rodriguez P, Gianola D, Gonzalez-Camacho JM, Crossa J, Manes Y, Dreisigacker S. Comparison between linear and non-parametric regression models for genome-enabled prediction in wheat. G3 (Bethesda). 2012;2:1595-605.
