# Supplementary material for: Impact of QTL number, heritability and reference population size on the benefit of machine learning models over GBLUP in genomic prediction
Source: Genet Sel Evol. 2026 Jul 13;58:49. doi: 10.1186/s12711-026-01065-6 (PMC13491627; doi:10.1186/s12711-026-01065-6)
Supplement: Supplementary file 2 [file 12711_2026_1065_MOESM10_ESM.docx]

Additional file 10:

Figure S1. Prediction accuracy of GBLUP, RF, and MLP-ResNet when heritability was 0.01. Box plots are shown as described in the caption of Fig. 3.

Figure S2. Difference in prediction accuracy between ML models and GBLUP when heritability was 0.01. Box plots are shown as described in the caption of Fig. 3.
