# Supplementary material for: Impact of QTL number, heritability and reference population size on the benefit of machine learning models over GBLUP in genomic prediction
Source: Genet Sel Evol. 2026 Jul 13;58:49. doi: 10.1186/s12711-026-01065-6 (PMC13491627; doi:10.1186/s12711-026-01065-6)
Supplement: Supplementary file 4 [file 12711_2026_1065_MOESM12_ESM.docx]

Additional file 12:

Table S1. Comparison of average correlation between predicted value of RF and TBV for training and test dataset for different scenarios

| Genetic settings | Reference population size | Cor (Predicted value_test, TBV) > that of train | Var (Predicted value_train) | Var (Predicted value_test) | Whether RF outperform GBLUP |
| --- | --- | --- | --- | --- | --- |
| 50 QTL, h2 = 0.1 | 1000 | Yes | 64.8 | 1.8 | Yes |
|  | 2000 | Yes | 17.3 | 1.0 | No |
|  | 10,000 | Yes | 7.0 | 3.0 | No |
|  | 50,000 | Yes | 19.6 | 2.8 | No |
| 50 QTL, h2 = 0.5 | 1000 | No | 11.5 | 1.0 | Yes |
|  | 2000 | No | 14.7 | 1.5 | No |
|  | 10,000 | No | 15.5 | 2.5 | No |
|  | 50,000 | No | 13.9 | 3.6 | No |
| 100 QTL, h2 = 0.1 | 1000 | No | 95.1 | 2.4 | No |
|  | 2000 | Yes | 136.2 | 2.2 | No |
|  | 10,000 | Yes | 7.0 | 2.0 | No |
|  | 50,000 | Yes | 7.3 | 3.4 | No |
| 100 QTL, h2 = 0.5 | 1000 | No | 10.3 | 1.6 | No |
|  | 2000 | No | 10.7 | 1.8 | No |
|  | 10,000 | No | 11.7 | 3.4 | No |
|  | 50,000 | No | 26.4 | 5.1 | No |
| 5000 QTL, h2 = 0.1 | 1000 | No | 2509.6 | 73.1 | No |
|  | 2000 | No | 1618.8 | 68.4 | No |
|  | 10,000 | Yes | 336.8 | 37.9 | No |
|  | 50,000 | Yes | 312.1 | 68.5 | No |
| 5000 QTL, h2 = 0.5 | 1000 | No | 643.7 | 24.5 | No |
|  | 2000 | No | 395.5 | 27.7 | No |
|  | 10,000 | No | 607.8 | 66.4 | No |
|  | 50,000 | No | 473.2 | 68.7 | No |

Table S2. Correlation between predicted value and TBV in training and test using TBV as input in one replicate*

|  | Correlation coefficient |
| --- | --- |
| Predicted value in training and TBV | 0.96 |
| Predicted value in test and TBV | 0.78 |

* QTL number = 50, heritability = 0.1, reference population size =2000, replicate 14
