# Supplementary material for: Impact of QTL number, heritability and reference population size on the benefit of machine learning models over GBLUP in genomic prediction
Source: Genet Sel Evol. 2026 Jul 13;58:49. doi: 10.1186/s12711-026-01065-6 (PMC13491627; doi:10.1186/s12711-026-01065-6)
Supplement: Supplementary file 5 [file 12711_2026_1065_MOESM13_ESM.docx]

Additional file 13:

Figure S1. Correlation between predicted values and residuals in training dataset of GBLUP, RF, SVR and MLP-ResNet at two heritability levels. Box plots are shown as described in the caption of Fig. 3.

Figure S2. Difference in prediction accuracy between MLP-ResNet and GBLUP across different scenarios.
