# Supplementary material for: Impact of QTL number, heritability and reference population size on the benefit of machine learning models over GBLUP in genomic prediction
Source: Genet Sel Evol. 2026 Jul 13;58:49. doi: 10.1186/s12711-026-01065-6 (PMC13491627; doi:10.1186/s12711-026-01065-6)
Supplement: Supplementary file 6 [file 12711_2026_1065_MOESM1_ESM.docx]

Additional file 1:

/*******************************

**	Global parameters	**

*******************************/

title = "Simulate selected populations";

nrep = 21; //Number of replicates

nthread = 5;	// Number of threads used

h2	= 0.5; //Heritability

qtlh2 = 0.0; //QTL heritability

phvar = 1.0; //Phenotypic variance

skip_inbreeding;

/*******************************

**	Historical population	**

*******************************/

begin_hp;

hg_size = 3000 [0] //Size of the historical generations

3000 [1200]	//

200 [1400]	// 200 or 250 animals

200 [1600]	// 200 or 250

10200 [1605];

nmlhg = 200; //Number of males in the last generation

end_hp;

/*******************************

**	Populations	**

*******************************/

begin_pop = "Currentpop";

begin_founder;

male [n = 200, pop = "hp", select = rnd];

female [n = 10000, pop = "hp", select = rnd];

end_founder;

ls = 2; //Litter size

pmp = 0.5 /fix; //Proportion of male progeny

ng = 16; //Number of generations

md = rnd; //Mating design

sr = 1;

dr = 1;

sd = ebv /h;

ebv_est = accur 0.80 /true_av;

begin_popoutput;

data /gen 11 12 13 14 15 16;

genotype /seq /gen 11 12 13 14 15 16 ;

stat;

end_popoutput;

end_pop;

/*******************************

**	Genome	**

*******************************/

begin_genome;

begin_chr = 10;

chrlen = 100; //Chromosome length

nmloci = 8000; //Number of markers

mpos	= rnd; //Marker positions

nma	= all 2; //Number of marker alleles

maf	= rnd; //Marker allele frequencies

nqloci = 0; //Number of QTL

qpos	= rnd; //QTL positions

nqa	= all 2; //Number of QTL alleles

qaf	= rnd;	//QTL allele frequencies

qae	= rndg 0.42; //QTL allele effects

end_chr;

r_mpos_g; //Randomize marker positions across genome

r_qpos_g; //Randomize QTL positions across genome

mean_crossover = 1;

mmutr = 0.00005 /recurrent;	//marker mutation rate

qmutr = 0.00005 /recurrent;	//qtl mutation rate

select_seg_loci; //maf in last historical generation

end_genome;

/*******************************

**	Output options	**

*******************************/

begin_output;

linkage_map;

hp_stat;

monitor_hp_homo;

end_output;
