# Supplementary material for: Impact of QTL number, heritability and reference population size on the benefit of machine learning models over GBLUP in genomic prediction
Source: Genet Sel Evol. 2026 Jul 13;58:49. doi: 10.1186/s12711-026-01065-6 (PMC13491627; doi:10.1186/s12711-026-01065-6)
Supplement: Supplementary file 7 [file 12711_2026_1065_MOESM2_ESM.docx]

Additional file 2:

Generation 11	Generation 12

Generation 13	Generation 14

Generation 15	Generation 16

Figure S1. Histogram of allele frequencies of 5000 informative QTL from generation 11 to 16 in one replicate

(a)	(b)

Figure S2. heritability of target trait for generation 11 to 16 across replicates when there were a) 100 QTL underlying the trait, b) 5000 QTL underlying the trait.
