# Supplementary material for: Impact of QTL number, heritability and reference population size on the benefit of machine learning models over GBLUP in genomic prediction
Source: Genet Sel Evol. 2026 Jul 13;58:49. doi: 10.1186/s12711-026-01065-6 (PMC13491627; doi:10.1186/s12711-026-01065-6)
Supplement: Supplementary file 8 [file 12711_2026_1065_MOESM3_ESM.docx]

Additional file 3:

Support vector regression (SVR) is a supervised learning method derived from support vector machines, designed to model the relationship between input features and the continuous response variable (Vapnik, 1995). Given a training set of $n$ samples ${\{\left( x_{i},y_{i} \right)\}}_{i=1}^{n}$, where $x_{i}\in\mathbb{R}^{d}$represents the genotype-based features (i.e. SNPs), and $y_{i}\in\mathbb{R}$ denotes the observed phenotypic value. $\mathbb{R}^{d}$ represents d-dimensional real space, and

SVR aims to find a function of the form:

$$f\left( x \right)= \left\langle w, \phi\left( x \right) \right\rangle+ b,$$

where $w\in\mathbb{R}^{d}$ is the weight vector, $b\in\mathbb{R}$ is the intercept term, and $\phi\left( x \right)$ denotes a transformation of the input data into a higher-dimensional feature space. The model seeks to minimize the following convex optimization problem:

$$\min_{w, b,\xi_{i},\xi_{i}^{*}} \frac{1}{2}{\parallel w\parallel}^{2}+C\sum_{i=1}^{n} (\xi_{i}+ \xi_{i}^{*}),$$

subject to the constraints:

$$\left\{ \begin{aligned} y_{i}-\left\langle w, \phi(x_{i}) \right\rangle-b\leq\varepsilon+\xi_{i} \\ \left\langle w, \phi(x_{i}) \right\rangle+b-y_{i}\leq\varepsilon+\xi_{i}^{*} \\ \xi_{i},\xi_{i}^{*}\geq0, i=1,\ldots,n \end{aligned} \right.$$

In this formulation, $\varepsilon$ defines an epsilon-insensitive zone, a concept unique to SVR. It defines a margin of tolerance around the true output within which the prediction is considered "good enough", and no penalty is applied for prediction error. $C>0$ is a regularization parameter that controls the trade-off between model complexity and tolerance to deviations outside the margin, and $\xi_{i},\xi_{i}^{*}$ are slack variables, which represent the extent to which a prediction deviates from the $\varepsilon$ margin. In our implementation, a radial basis function (RBF) kernel was used to allow for non-linear modeling of genotype-phenotype relationships, defined as:

$$K\left( x_{i}, x_{j} \right)= exp\left( -\gamma{\parallel x_{i}-x_{j}\parallel}^{2} \right)$$

where $\gamma>0$ is a kernel parameter that controls the smoothness of the function.
