# Supplementary material for: Impact of QTL number, heritability and reference population size on the benefit of machine learning models over GBLUP in genomic prediction
Source: Genet Sel Evol. 2026 Jul 13;58:49. doi: 10.1186/s12711-026-01065-6 (PMC13491627; doi:10.1186/s12711-026-01065-6)
Supplement: Supplementary file 9 [file 12711_2026_1065_MOESM7_ESM.docx]

Additional file 7:

Table S1. Computation time (minutes) of different models for replicate 1 with a reference population size of 50,000 (1 CPU)

| Models | Heritability | | | | | | Average |
| --- | --- | --- | --- | --- | --- | --- | --- |
|  | 0.1 | | | 0.5 | | |  |
|  | QTL number | | | | | |  |
|  | 50 | 100 | 5000 | 50 | 100 | 5000 |  |
| GBLUP* | 645 | 602 | 486 | 299 | 315 | 384 | 455 |
| RF | 101 | 82 | 520 | 169 | 146 | 98 | 186 |
| SVR | 584 | 604 | 631 | 4169 | 3679 | 6572 | 2706 |
| MLP-ResNet | 55 | 42 | 16 | 80 | 56 | 48 | 50 |

***** Not included time to construct G matrix using calc_grm.

Table S2. Simulation settings with different optimal hyperparameter combinations and the computation time for replicate 1 of SVR with a reference population size of 50,000 (1 CPU)

|  | Hyperparameters | | | Computation time (min) |
| --- | --- | --- | --- | --- |
|  | C | ε | γ |  |
| 50 QTL, h2 = 0.1 | 0.1 | 0.5 | scale | 584 |
| 50 QTL, h2 = 0.5 | 0.1 | 0.5 | scale | 4169 |
| 100 QTL, h2 = 0.1 | 1 | 0.5 | scale | 604 |
| 100 QTL, h2 = 0.5 | 1 | 0.1 | scale | 3679 |
| 5000 QTL, h2 = 0.1 | 1 | 0.5 | scale | 631 |
| 5000 QTL, h2 = 0.5 | 10 | 0.5 | scale | 6572 |
