# Supplementary material for: Impact of QTL number, heritability and reference population size on the benefit of machine learning models over GBLUP in genomic prediction
Source: Genet Sel Evol. 2026 Jul 13;58:49. doi: 10.1186/s12711-026-01065-6 (PMC13491627; doi:10.1186/s12711-026-01065-6)
Supplement: Supplementary file 10 [file 12711_2026_1065_MOESM9_ESM.docx]

Additional file 9:

Table S1. Results of MLP-ResNet and GBLUP for one replicate*

|  | MLP-ResNet | | | MLP-ResNet (Tuned) | GBLUP |
| --- | --- | --- | --- | --- | --- |
| Random seed value | 8 | 16 | 42 | 16 |  |
| Accuracy | 0.400 | 0.019 | 0.378 | 0.411 | 0.425 |
| Dispersion bias | 1.34 | 0.21 | 2.37 | 1.31 | 1.22 |

*50 QTL, h^2^ = 0.1, reference population size = 1000, replicate 8
